# Sonosensitive MRI Nanosystems as Cancer Theranostics: A Recent Update

**DOI:** 10.3389/fchem.2018.00157

**Published:** 2018-05-07

**Authors:** Francesca Garello, Enzo Terreno

**Affiliations:** Molecular and Preclinical Imaging Centers, Department of Molecular Biotechnology and Health Sciences, University of Torino, Torino, Italy

**Keywords:** sonosensitive agents, image-guided drug release, ultrasounds, magnetic resonance imaging, theranostics, thermosensitive liposomes, HIFU, LINFU

## Abstract

In the tireless search for innovative and more efficient cancer therapies, sonosensitive Magnetic Resonance Imaging (MRI) agents play an important role. Basically, these systems consist of nano/microvesicles composed by a biocompatible membrane, responsive to ultrasound-induced thermal or mechanical effects, and an aqueous core, filled up with a MRI detectable probe and a therapeutic agent. They offer the possibility to trigger and monitor in real time drug release in a spatio-temporal domain, with the expectation to predict the therapeutic outcome. In this review, the key items to design sonosensitive MRI agents will be examined and an overview on the different approaches available so far will be given. Due to the extremely wide range of adopted ultrasound settings and formulations conceived, it is hard to compare the numerous preclinical studies reported. However, in general, a significantly better therapeutic outcome was noticed when exploiting ultrasound triggered drug release in comparison to traditional therapies, thus paving the way to the possible clinical translation of optimized sonosensitive MRI agents.

## Introduction

New insights in cancer biology, along with the advances in early detection and treatment, led to increased life expectancy, and reduced cancer related deaths. However, there is still a strong need for more efficient, precise, and safer therapies. To decrease the systemic toxicity of some chemotherapeutic agents, drug encapsulation into biocompatible nanovesicles, named liposomes, has been envisaged. The FDA approval of Doxil®/Caelyx® in 1995, a doxorubicin liposomal formulation, for the treatment of Kaposi sarcoma, metastatic breast cancer and recurrent ovarian cancer (Barenholz, 2012), boosted the research for optimizing liposomal preparations. Liposomes are highly versatile nanovesicles consisting of a phospholipid bilayer surrounding an aqueous core; these vesicles can vary in size, shape, and lipid composition (Sessa and Weissmann, 1968). Basically, liposomes can carry drugs and/or other molecules, such as imaging or targeting agents, both in their aqueous core and/or in the lipidic membrane.Liposomes are designed to prevent drug extravasation into healthy tissues, prolong blood circulation time, improve drug accumulation, and enhance bioavailability at the target site (Blanco et al., 2015). The accumulation of liposomes into solid tumors is mostly due to peculiarities of the cancerous tissue, namely increased blood supply, enhanced endothelial permeability and reduced lymphatic drainage, resulting into the Enhanced Permeability and Retention (EPR) effect (Maeda et al., 2000).

Once accumulated in the tumor, the drug needs to be released from the carrier. For the nanomedicines currently approved in clinics, this crucial step occurs spontaneously, i.e., following the natural degradability of the nanocarrier interacting with tissue components (Rizzitelli et al., 2015). However, to better control this process, liposomes sensitive to endogenous (e.g., pH, redox potential, enzymatic activity) or exogenous (e.g., heat, light, pressure) stimuli can be designed (Guo and Szoka, 2003). Among different possibilities, in the last decade, considerable attention has been devoted to the use of ultrasounds (US) (Pong et al., 2006), as they are already clinically approved tools for imaging and therapy. Moreover, they can be modulated in terms of frequency and intensity according to the specific goal to be achieved. In addition, in view of personalized medicine based approaches, co-encapsulation of an imaging agent inside the vesicle could be envisaged in order to follow the release process and predict the therapeutic outcome. In this respect, Magnetic Resonance Imaging (MRI), due to its outstanding spatial resolution, low invasiveness and absence of limits in tissue penetration could be regarded as the optimal imaging technique. Small paramagnetic molecules can be loaded into liposomes in order to spatio-temporally track drug delivery and/or drug release. In the next paragraphs an overview on the different sonosensitive MRI agents will be presented, with a focus on ultrasound-based trigger mechanisms. Finally, some examples of successful preclinical applications in this field will be reported.

## Ultrasound triggered drug release

Ultrasound is a form of mechanical energy characterized by an acoustic pressure wave at frequencies beyond the upper limit of the normal human sound range, which is from 20 to 20,000 Hz. Basically, US are produced by a sound source vibrating sinusoidally along time, back and forth in space (Xin et al., 2016). In practice US are often produced by means of a ceramic disk endowed with a piezoelectric effect and a specific radius. The disk is inserted within a transducer linked to a waveform function generator. The produced ultrasounds vary in frequency, amplitude, intensity, and speed of propagation. Frequency is the number of cycles of compressions and rarefactions in a sound wave per second. Ultrasounds can be classified according to frequency into low (20–200 kHz), medium (0.7–3.0 MHz), or high (1–20 MHz) frequency waves. Low frequency US are characterized by deeper tissue penetration. Moreover, US can be further grouped according to intensity, defined as the quantity of energy in the US beam area, into low intensity (<3 W/cm^2^) or high intensity ultrasound (3–10,000 W/cm^2^) (ter Haar, 1999). Low intensity ultrasounds are already clinically approved to promote transdermal drug delivery, while high intensity US are mainly used for thermal ablation of uterine fibroids, kidney stone shattering, and palliative treatments. The possibility of using pulsed or continuous waves, over a variable time range, as well as focusing US by means of specific transducers, makes this technique extremely versatile. Ultrasounds can induce drug release in two different ways, namely by the mechanical or thermal route.

### US mechanical effect

The US mechanical effect is based upon a combination of micromassage, cavitation and acoustic streaming. Micromassage refers to US induced cell vibrations, likely affecting tissue fluid interchange and tissue mobility. Cavitation is defined as the phenomena of the formation, growth, and subsequent collapse of microbubbles (Frenkel, 2008). Newly formed microbubbles or administered sonosensitive vesicles can oscillate stably (stable cavitation), inducing a constant fluid flow around the bubble, called microstreaming, that induces stress on cell membranes, and may enhance cell permeability. While, if the bubbles increase more than twofold their size, they violently collapse (inertial cavitation) causing microstreaming, formation of liquid jets and ultrasound shock wave emission, able to disrupt membranes of adjacent cells and create pores in capillary walls (Khokhlova et al., 2015). As cavitation phenomena can induce severe cytotoxic effects, a cavitation level sufficient to release drug form nanovesicles and permeabilize cell membranes but without killing cells should be induced (Pitt et al., 2004). To exploit this wide range of mechanical effects, specific US responsive nano-microsystem have been designed, mainly microbubbles (Hernot and Klibanov, 2008), and liposomes (Schroeder et al., 2007). More details about these systems are provided in section Sonosensitive Systems.

### US thermal effect

The thermal effect, recently reviewed by T. Boissenot et al. (2016), is strictly linked to the application of high intensity US. More in details by focusing the US beam in a small area of a target tissue, the power per cross section area becomes extremely high, leading to significant absorption of thermal energy from the beam by the tissue and consequently resulting in local temperature rise. Resulting hyperthermia could be mild (39–42°C) or high (>43°C). Mild hyperthermia is generally employed to trigger drug release, as it will be discussed in paragraph Thermosensitive Systems. Whereas, high hyperthermia is mainly exploited to kill or ablate tissues (Diederich and Hynynen, 1999): uterine fibroids, prostate, breast, pancreatic, and liver cancers have been safely and successfully treated with High Intensity Focused Ultrasound (HIFU). In this regard, precise, and constant temperature monitoring of the heated area is of paramount importance; it can be obtained by means of invasive thermocouples or by MRI thermometry.

## Ultrasound responsive and MRI detectable probes

**Figure 1:**
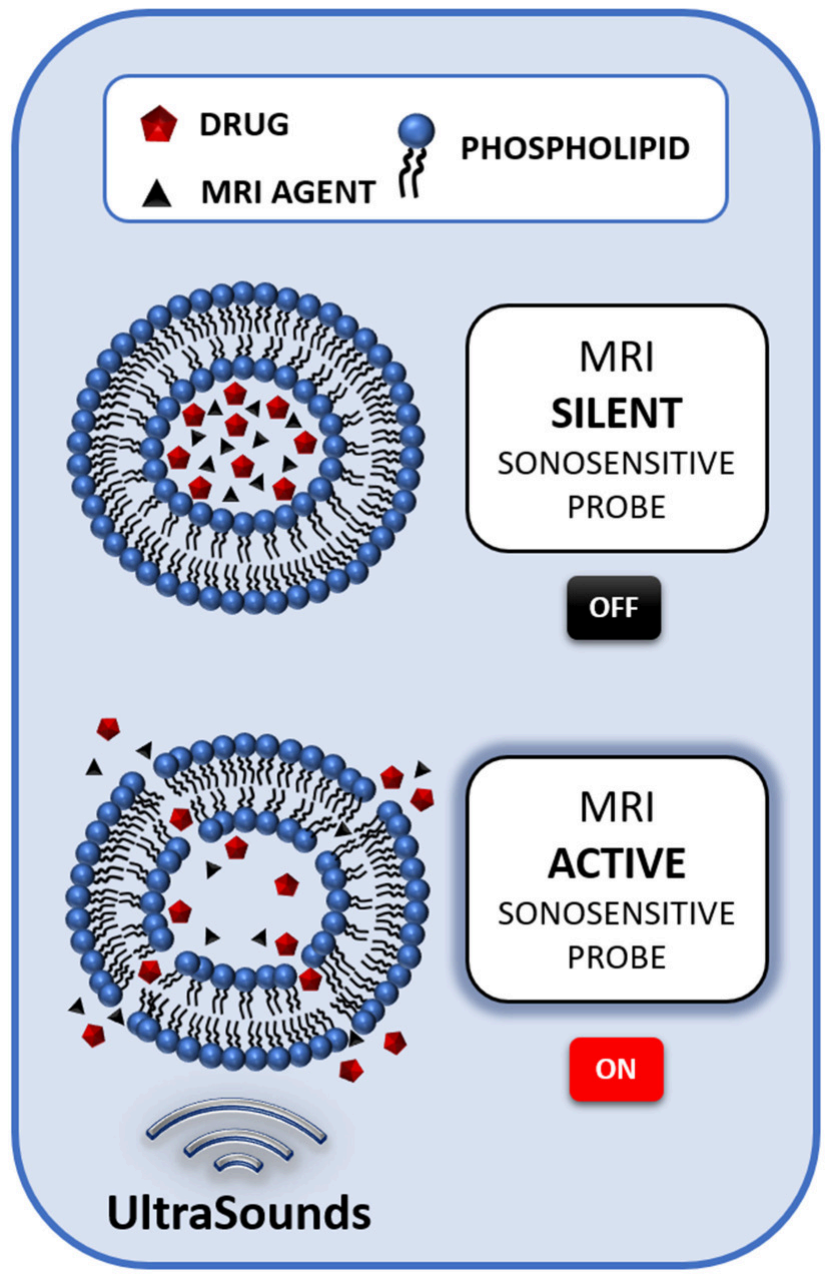
Schematic illustration of the mechanism of a liposome-based sonosensitive MRI agent. The MRI signal of a low water permeable liposome entrapping a relatively high amount of a MRI agent is almost silent, due to the compartmentalization of the probe. However, when the US stimulation triggers the release of the agent, the MRI contrast activates, thus signaling the release of the drug co-loaded in the nanocarrier.

So far, a number of reports has been published about the synthesis and optimization of ultrasound responsive and MRI detectable probes. The key components to design such systems are: (i) a lipidic membrane stable in physiological conditions, able to release its content selectively upon US exposure; (ii) a MRI agent, encapsulated within the system, able to report on drug release; (iii) an entrapped drug, that, upon release, carries out the therapeutic effect (Figure 1).

### Sonosensitive systems

Initially invented as contrast agent for ultrasound imaging and lately reconsidered for drug and gene delivery purposes, microbubbles are micron sized systems made of an external shell encapsulating an inner gas core. These systems display a strong tendency to undergo stable or inertial cavitation upon US exposure. The shell could be made up of lipids, polymers or proteins. The inner core could also be filled with liquid perfluoropropane, then triggered to the gaseous phase when stimulated with acoustic energy. Theranostic microbubbles were prepared by Fan et al. bearing a phospholipidic shell, endowed with a complex of SuperParamagnetic Iron Oxide (SPIO) nanoparticles and doxorubicin, and filled with perfluoropropane (Fan et al., 2016). Similar, nanosized, probes were developed by Cavalli et al. (2015). Palmitic acid was the surfactant of choice to be included in the shell in order to entrap the MRI agent Gd-DOTP. Prednisolone phosphate (PLP) was added as therapeutic agent, while pluronic F68 was used as stabilizing agent. Both the preparations, however, were characterized by limited stability over time (1–3 h).

To this regard, liposomes appear promising systems, but, in order to be sonosensitive, special attention has to be dedicated to their membrane composition. TJ Evjen (2011) highlighted the important role played by the phospholipid Packing Parameter (PP)_._ Non-bilayer forming lipids with large hydrophobic cross section as compared to the polar headgroup (PP >1), like DOPE or DSPE, significantly promote liposome sonosensitivity (Evjen et al., 2013). This effect is associated to a DOPE/DSPE restructuring process: upon US exposure, a transformation from lamellar to reversed hexagonal phase occurs, inducing the formation of pores and/or tubular aggregates, through which the drug can readily leak out (Kang et al., 2014). In order to form liposomes, DOPE (or other phospholipids bearing PP < 1) must be mixed with phospholipids displaying natural tendency to form bilayers (PP~1), cholesterol, useful to induce mechanical stability by tightening the membrane, and stealth moieties, to prolong blood circulation time. While the amount of cholesterol, saturated phospholipids, and polymers should be limited as they increase membrane stiffness and decrease the tendency to drug leakage, stealth moieties, like DSPE-PEG2000 seems to enhance sonosensitivity by the so called “antenna effect,” favoring the interactions between acoustic waves and the vesicles. Finally, Giustetto et al. demonstrated that also shape, size, and intravesicular composition of liposomes may influence US triggered drug release (Giustetto et al., 2013).

### Thermosensitive systems

In 1978, Yatvin et al. first described a thermosensitive formulation, consisting of a DPPC/DSPC (3:1) liposome able to selectively release its hydrophilic content when the temperature was raised up of a few degrees above physiological temperature (Yatvin et al., 1978). Since then, many efforts have been made to obtain improved formulations and to accurately monitor temperature variations in the region of interest, where drug release should take place. The fundamental points to be fulfilled to design a good thermosensitive system are:(i) the presence of temperature sensitive phospholipids or polymers in the membrane; (ii) stable encapsulation of drugs at body temperature; (iii) fast and complete drug release upon heat stimulation and (iv) provision of high drug plasma levels during the time span of hyperthermia treatment (Hijnen et al., 2014). The first thermosensitive liposomes (TSL) developed were mainly composed of phosphatidylcholines, bearing a transition temperature (T_m_) in the range of mild hyperthermia (40–43°C). Around T_m_, the temperature at which a polymer/phospholipid melts from the gel-ordered phase to the liquid-crystalline phase, a significant drug release was achievable due to grain boundaries between the two phases (Landon et al., 2011). In 1994 Unezaki et al. (1994) added 3% PEGylated phospholipids to a DPPC/DSPC 9:1 formulation, resulting in prolonged circulation time, due to PEG stealth effect, and better therapeutic outcome. PEGylated phospholipids were then considered a basic component.

A breakthrough occurred in 2000, when Needham and co-workers proposed the inclusion of lysolipids (PP < 1) into the membrane bilayer (DPPC/MPPC/DSPE-PEG2000 90:10:4), obtaining 80% drug release around T_m_ in a few seconds, thanks to the formation of lysolipids-stabilized membrane pores (Needham et al., 2000). This innovation led to the birth of ThermoDox®, a TSL formulation containing lysolipids and doxorubicin, that has reached phase III clinical trials for hepatocellular carcinoma treatment (Bulbake et al., 2017). In recent years a myriad of subtle variations has been made to the above-mentioned formulations and different simulated or experimental models have been developed to predict the complex interplay between liposome properties, tumor perfusion, heating regimen, and therapeutic efficacy (Gasselhuber et al., 2010, 2012; Lokerse et al., 2016, 2017). Interestingly, Banno et al. drew the attention on the importance of TSL formulation to retain liposome stability: they reported that 70% of lysolipids was lost within 1 h post injection of TSL, likely due to interactions with the large lipid membrane pool *in vivo*, thus inducing non-triggered drug leakage (Banno et al., 2010). The advantage of using HIFU as heating source is the possibility to obtain a fast and localized temperature increase. Moreover, spatial guidance and temperature monitoring are nowadays available thanks to non-invasive MR-HIFU systems. Among different magnetic resonance thermometry techniques available, Proton Resonance Frequency Shift (PRFS) is by far the most employed, with a precision of approximately 1°C. Using the MR-HIFU platform different focal points can be steered, heating tissues of various volumes, meanwhile sparing vulnerable and crucial structures (Hijnen et al., 2014). It has to be mentioned that MR-thermometry could be perturbed by MRI contrast agents loaded into TSL, due to relaxivity changes induced by the agent accumulation. Conception of correction methods is therefore urgently needed. To overcome this problem, recently Shin et al. proposed the use of perfluorocarbon nanoemulsions as ^19^F MR contrast agent in sonosensitive systems, in order not to have interferences with ^1^H based PRFS (Shin et al., 2017). However, it seems quite challenging to obtain temporal information about drug release using fluorinated compounds.

### MRI reporter agents

Co-encapsulation of MRI agents into sonosensitive nanovesicles, thus obtaining sonosensitive MRI agents, is of paramount importance in order to monitor and assess in real-time the drug release process. The most diffuse approach consists in encapsulating small hydrophilic paramagnetic compounds, based on Gd^3+^ or Mn^2+^ ions, in the aqueous core of the nanovesicles. Upon the entrapment the MRI contrast is “silenced,” due to reduced exchange rate of water molecules across the liposomal membrane. When the agent is released, the “quenching effect” is removed, allowing the detection of a contrast enhancement. This contrast enhancement can be estimated and used as reporter of the extent of drug delivery (Ponce et al., 2007; de Smet et al., 2011; Tagami et al., 2011; Rizzitelli et al., 2016). The first TSL encapsulating a MR contrast agent was developed by Viglianti et al. who co-loaded MnSO_4_ and doxorubicin in liposomes and detected a change in relaxivity upon heat-triggered drug release (Viglianti et al., 2004). De Smet et al. instead, entrapped into TSL both the clinically approved contrast agent Gd-HPDO3A and doxorubicin, demonstrating that: (i) the paramagnetic compound did not affect doxorubicin loading and release, (ii) the drug and the imaging agent were released simultaneously upon heating and (iii) Gd^3+^ encapsulation within the aqueous core quenched its relaxivity until release occurred (de Smet et al., 2010). However, in both works release was obtained with non-US mediated hyperthermia. Only one year later, the system conceived by de Smet et al. was exploited for MR-HIFU triggered drug release by Negussie et al. co-workers, who demonstrated that upon HIFU stimulation the releases of the drug and the imaging agent were comparable (Negussie et al., 2011), thus paving the way to real-time drug release estimation by MRI using the paramagnetic compound as doxorubicin surrogate. Similar conclusions were drawn by Tagami et al. who co-encapsulated Gd-DTPA and doxorubicin (Tagami et al., 2011), and Rizzitelli et al. who demonstrated the feasibility of stimulating with pulsed Low Intensity Non Focused Ultrasounds (pLINFU) and tracking by MRI drug release from nanovesicles doped with gadoteridol and doxorubicin (Rizzitelli et al., 2014). In 2013, Han et al. developed a sonosensitive system loaded with doxorubicin and endowed of a newly-synthesized Gd-DOTA-DPPE lipid. These vesicles were intended to track drug delivery by MRI, but were unable to report on drug release as Gd^3+^ was incorporated in the vesicle membrane, thus preventing the removal of the so-called “quenching effect” (Han et al., 2013). Original approaches included the use of a dysprosium chelate (Fowler et al., 2013) or Chemical Exchange Saturation Transfer (CEST) contrast agents. Langereis and co-workers loaded into TSL both a chemical shift agent (Tm-HPDO3A, for ^1^H lipoCEST detection) and a highly fluorinated compound (hexafluoro-phosphate, for ^19^F detection). When the two agents were compartmentalized, liposomes could be visualized solely by the CEST effect due to the influence of the paramagnetic shift agent exerted over ^19^F-NMR resonance, but once the release was triggered the lipoCEST contrast enhancement vanished, while the ^19^F MRI signal appeared, allowing release quantification (Langereis et al., 2009). Delli Castelli et al. entrapped Eu-HPDO3A in ultrasound-sensitive liposomes, resulting in a “quenched” paraCEST effect at 18 ppm, promptly restored following disruption of the liposomal membrane (Delli Castelli et al., 2014). Finally, iron oxide based contrast agents have been employed. For instance, it has been shown that sonosensitive liposomes coated with hydroxyapatite and entrapping nanodots of SPIO in the inorganic shell can release their content upon application of ultrasounds, inducing changes in ${\text{T}}_{2}^{*}$/T_2_ MRI contrast (Liu and Huang, 2011). Lorenzato et al. developed temperature-sensitive magnetoliposomes, encapsulating ultrasmall iron oxide particles (USPIO), displaying significant differences in MRI signal enhancement and relaxivities before and after HIFU stimulation (Lorenzato et al., 2013). In the next paragraph the most promising preclinical applications of sonosensitive MRI agents will be reported.

## Preclinical studies

A direct comparison of the performance of preclinical studies involving sonosensitive MRI agents is not an easy task as they vary not only in terms of liposomal formulation but also in the type of ultrasounds employed to trigger the release (Table 1). Basically, we can gather these works into three groups: (i) HIFU exploited to induce hyperthermia to stimulate both the release of the imaging agent and tissue ablation (no drugs encapsulated); (ii) HIFU employed to trigger the release of both the imaging and the therapeutic agent by hyperthermia; (iii) pLINFU used to trigger the release of the drug and the imaging moiety by mechanical effect. A representative example of the first group is the work reported by McDannold et al. dealing with non-stealth TSL loaded with Gd(DTPA-BMA). The liposomes were injected in rabbits bearing VX2 liver tumors for MR monitoring of thermal therapy carried out with HIFU, obtaining a good match to traditional MR thermometry methods only in the liver (McDannold et al., 2004). The aim was to provide a less motion-sensitive technique to monitor temperature in real time.

**Table 1:** Overview of preclinical studies performed with sonosensitive MRI agents.

**Referencess**	**LIPOSOMAL FORMULATION**	**MRI PROBE**	**DRUG**	**DISEASE MODEL**	**US SETUP**
McDannold et al., 2004	DSPC:DSPG (90:10 w/w)	Gd(DTPA-BMA)	No	Liver VX2 tumors (*rabbit*)	HIFU 1.71 MHz
Negussie et al., 2011	DPPC:MSPC:DSPE-PEG_2000_ (85.3:9.7:5.0 mol/mol)	Gd(HPDO3A)	Doxorubicin	VX2 tumor in thigh muscle (*rabbit*)	HIFU 1.2 MHz
de Smet et al., 2011	DPPC:HSPC:Chol:DPPE-PEG_2000_ (50:25:15:3 mol/mol)	Gd(HPDO3A)	Doxorubicin	Subcutaneous 9L gliosarcoma tumors (*rat*)	HIFU 1.4 MHz
de Smet et al., 2013	DPPC:HSPC:Chol: DPPE-PEG_2000_:DOTA-DSPE (50:25:15:3:1 mol/mol)	Gd(HPDO3A)	Doxorubicin	R1 rhabdomyosarcoma tumors (*rat*)	HIFU 1.4 MHz
Fowler et al., 2013	DSPE:DSPE-PEG_2000_:Chol (62:8:30 mol/mol)	Gd(DTPA-BMA) Dy(DTPA-BMA)	No	Subcutaneous prostatic adenocarcinoma (*rat*)	Pulsed HIFU MHz PRF 250 Hz
Rizzitelli et al., 2016	DPPC:DSPC:Chol:DSPE-PEG_2000_ (10:5:4:1 mol/mol)	Gd(HPDO3A)	Doxorubicin	Subcutaneous TSA Breast Cancer (*mouse*)	Pulsed LINFU 1 MHz/3 MHz PRF 1 Hz
Fan et al., 2016	DSPC:DSPG:DSPE-PEG_2000_ (21:21:1 mol/mol)	Superparamagnetic iron oxide (SPIO)	Doxorubicin	C6 glioma (rat)	FU 1 MHz
Hijnen et al., 2017	DPPC:HSPC:Chol:DPPE-PEG_2000_ (50:25:15:3 mol/mol)	Gd(HPDO3A)	Doxorubicin	R1 rhabdomyosarcoma tumors (*rat*)	HIFU 1.44 MHz

*HIFU, High Intensity Focused Ultrasounds; LINFU, Low Intensity Non Focused Ultrasounds; FU, Focused Ultrasounds*.

Several works report on the design and testing of TSLs loaded with doxorubicin and Gd^3+^ complexes, to track drug release induced by US thermal effect (de Smet et al., 2011; Negussie et al., 2011). In the study reported by De Smet et al. TSL coencapsulating doxorubicin and Gd-HPDO3A were administered to rats bearing subcutaneous 9L gliosarcoma tumors. Local hyperthermia (42°C) was applied for 30 min through HIFU and drug release was monitored with interleaved T_1_ mapping of the tumor tissue, finding out a good correlation between released doxorubicin and Gd^3+^ (de Smet et al., 2011). In 2016 Lokerse et al. seeking for an optimized Dox-TSL formulation, found that the prediction of liposomal efficiency based merely on *in vitro* test is challenging (Lokerse et al., 2016). One year later Hijnen et al. compared different MR-HIFU treatment schemes, assessing that a combination protocol of hyperthermia-induced drug delivery with TSL, followed by ablation, resulted in a homogeneous drug distribution and in the highest therapeutic effect (Hijnen et al., 2017).

The only preclinical study using pLINFU was published by Rizzitelli et al. In this work, Gd-HPDO3A was loaded in a Doxil®-like preparation and administered to mice bearing subcutaneous breast cancer. The most relevant aspect of this study dealt with the achievement of complete tumor regression following the injection of liposomes and the application of pLINFU for drug release and cell membrane sonoporation. Worth of note is the fact that the used theranostic agent is made of already clinically approved agents (Doxil®, Gd-HPDO3A). The authors outlined that this approach may offer the possibility of predicting the therapeutic outcome in each patient, simply looking at the MRI contrast enhancement 15 min *p.i*. (Rizzitelli et al., 2016). The latter aspect, also known as “dose painting,” is of crucial importance in view of a more and more personalized medicine.

## Conclusion

This minireview underlines the novelty and the potentiality of the topic, as most of the sonosensitive agents were developed in the last few years. Chemistry plays a fundamental role in the selection of the lipids composing the membrane, in order to boost drug release under US stimuli, while stably trapping the content under physio-pathological conditions in the absence of an external stimulus. It is stressed how special attention has to be devoted to the choice of the encapsulated MRI active compound, that has to act as a quantitative reporter of drug release. Regrettably, making critical comparison is rather difficult at present, due to the lack of standardization of transducers and circuits, often highly customized. However, even if a standardization of the US setting protocol is still lacking, mathematical and practical models are under development in order to predict the efficiency of the various formulations, and the obtained *in vivo* results appear very promising for a future clinical translation.

## Author contributions

FG organized the database and wrote the first draft of the manuscript; ET contributed to the conception and planning of the review, and revised the work critically for important intellectual content. All authors contributed to manuscript revision, read, and approved the submitted version.

### Conflict of interest statement

The authors declare that the research was conducted in the absence of any commercial or financial relationships that could be construed as a potential conflict of interest.

## Abbreviations

CEST: Chemical Exchange Saturation Transfer
DOPE: 1,2-dioleoyl-sn-glycero-3-phosphoethanolamine
DPPE: 1,2-Dipalmitoyl-sn-glycero-3-phosphoethanolamine
Gd-DOTP: Gadolinium-1,4,7,10-tetraazacyclododecane-N,N',N”,N”'-tetrakis(methylenephosphonic acid)
Gd-DTPA: Gadolinium-diethylenetriamine pentaacetic acid
Gd-DTPA-BMA: Gadolinium-di-ethylenetriaininepentaacetic acid-bis(methylamide)
Ln-HPDO3A: Lanthanide-1,4,7-triscarboxymethyl-1,4,7,10-tetraazacyclododecane
DSPE: 1,2-distearoyl-sn-glycero-3-phosphoethanolamine
DSPE-PEG2000: 1,2-distearoyl-sn-glycero-3-phosphoethanolamine-N-[methoxy(polyethylene glycol)-2000] (ammonium salt)
DPPC: 1,2-dipalmitoyl-sn-glycero-3-phosphocholine
DSPC: 1,2-distearoyl-sn-glycero-3-phosphocholine
HIFU: High Intensity Focused Ultrasound
MPPC: 1-palmitoyl-2-hydroxy-sn-glycero-3-phosphocholine
MRI: Magnetic Resonance Imaging
NMR: Nuclear Magnetic Resonance
pLINFU: Pulsed Low Intensity Non Focused Ultrasounds
PP: Packing Parameter
PRFS: Proton Resonance Frequency Shift
SPIO: SuperParamagnetic Iron Oxide
**T**_m_: Transition Temperature
TSL: Thermosensitive Liposomes
US: Ultrasounds
USPIO: Ultrasmall Iron Oxide Particles.
